# Effects of birth weight on body composition, physical fitness, and sarcopenia assessments in young Japanese women

**DOI:** 10.1371/journal.pone.0297720

**Published:** 2024-09-11

**Authors:** Tomohiro Yasuda

**Affiliations:** School oeef Nursing, Seirei Christopher University, Hamamatsu, Japan; Ehime University Graduate School of Medicine, JAPAN

## Abstract

This study examined the effects of birth weight on body composition, physical fitness, and sarcopenia in adulthood among young Japanese women. Seventy young adult women (birth weight <2500 g classified as low-birth-weight group [L-BW, *n* = 13] and ≥2500 g classified as not low-birth-weight group [NL-BW, *n* = 57]) were evaluated for body composition, physical fitness, and sarcopenia. Skeletal muscle mass was significantly greater (*p*<0.05) in the NL-BW group than in the L-BW group for all body sites. Effect sizes for the differences in skeletal muscle mass between the two groups were all larger in the NL-BW group than in the L-BW group (0.86–1.44). Knee extension muscle strength was higher in the NL-BW group than in the L-BW group (*p* = 0.04), but there were no differences between groups with respect to other physical fitness indicators (*p*>0.05). Except for SMI (*p*<0.05), other sarcopenia diagnostic evaluations did not differ between the two groups (*p*>0.05). In conclusion, L-BW female infants were shorter in standing height and smaller in skeletal muscle mass in terms of morphology at the time of young adulthood compared to NL-BW female infants. In addition, in terms of physical fitness, those with L-BW also had a lower-limb power score and a higher proportion of low skeletal muscle mass. Thus, it was suggested that low birth weight infants may be at risk of needing nursing care in old age (i.e., a high predicted incidence of sarcopenia) as well as thinness problems in the fertile generation.

## Introduction

In Japan, the percentage of low-birth-weight babies has been increasing year by year since the 1980s and has remained at approximately 10% in recent years without any decreasing trend [1–3]. Similarly, the percentage of underweight young adult women aged 20–29 has also increased since approximately 1980, and approximately 20% of young adult women are presently reported to be underweight [4,5].

Recently, it has been reported that women of reproductive age in Japan have a strong desire to be thin [6,7], and it is thought that the desire to be thin among young adult women has encouraged an increase in underweight among the fertile generation, resulting in an increase in low-birth-weight infants. Birth weight has been reported to be related to grip strength or aerobic fitness in middle age (men and women in their 30s or 50s [8,9], suggesting that birth weight may have a significant impact on physical morphology and fitness in the following decades of life.

On the other hand, the relationship with birth weight is often reported from the perspective of "middle age" or "muscle strength," and it remains unclear whether it also affects body composition and physical fitness in young adulthood. It would be very useful for early care prevention (sarcopenia prevention) if it were known whether low-birth-weight infants are affected in young adulthood and if the effects extend to physical morphology.

Although the relationship with birth weight has not been investigated, Yasuda [10] reported that many young Japanese women have problems with low skeletal muscle mass, and it has recently been noted that thinness in young adult women may be related to future sarcopenia (age-related muscle loss). Therefore, it is important to examine the morphology and physical fitness of young adults who were low-birth-weight infants and to perform sarcopenia assessments for future health problems. Thus, the present study examined the effects of birth weight on body composition, physical fitness and sarcopenia in adulthood among young Japanese women. This study provides important implications for appropriate health awareness, especially for the fertile generation.

## Methods

### Participants

Healthy young women were verbally recruited to participate in the study, and 70 Japanese women between the ages of 18 and 20 were registered. In Japan, individuals over the age of 18 are legally defined as adults, and minors were not included in this study. All participants had no chronic diseases (e.g., angina, myocardial infarction, diabetes, cancer, or stroke) evaluated at their annual physical examinations, and none had a history of musculoskeletal disease or knee surgery. Prior to obtaining informed consent, a written document explaining the purpose and safety of the study and a lifestyle questionnaire were distributed to potential participants. Participants in the study were classified as recreationally active, with 47 of the 70 participants exercising regularly (walking, jogging, or cycling for approximately 30 minutes two to three times a week) and eight belonging to an athletic club. All participants who passed the criteria were included in the data analysis. Participants were divided into the following two groups according to birth weight (BW): based on the World Health Organization (WHO) standards [11], a birth weight of less than 2500 g was defined as the low BW (L-BW) group, and a birth weight of 2500 g or more was defined as the not low BW (NL-BW) group. The principles of the Medical Association Declaration of Helsinki guidelines for the use of human participants were adopted for this study. This research was approved by the Ethics Committee of Seirei Christopher University, and written informed consent was obtained from all participants. The recruitment period for this study began on June 7, 2022, and the experiment ended on March 10, 2023.

### Physical fitness evaluation

Maximum voluntary isometric contraction of the knee extensors was measured using a knee extension dynamometer (TKK 5710, TAKEI, Tokyo, Japan). Participants sat in a chair positioned with the hip joint at an angle of approximately 85° (full extension = 0°). The ankle of the participant’s right leg was firmly secured to the strain gauge transducer with a chain strap (TKK 5002, TAKEI, Tokyo, Japan). After a warm-up consisting of a submaximal contraction immediately prior, participants were instructed to demonstrate maximal knee extension with the knee joint angled at approximately 90° (2–3 seconds). All participants performed two trials, with the highest value of each trial used for analysis [12].

All participants were instructed to perform a vertical jump and a standing broad jump. Participants were given standard instructions that allowed them to start the jump with a full knee bend and then swing their arms to assist in the jump. For the vertical jump, each participant stood with feet on the ground and jumped to the highest position with arms and hands extended. A sensor (T2290, TOEILIGHT, Saitama, Japan) strapped to the participant’s waist measured the distance the participant jumped upward from the starting position (standing height). Vertical jump height was measured in 1 cm increments, and the maximum value obtained in two trials was used as the vertical jump score. For the standing broad jump, a 3-m-long rigid mat (T2598, TOEILIGHT, Saitama, Japan) was used. The length of the jump was measured using a drawn line and a tape measure. The distance from the line to the landing point of the heel closest to the starting line was measured in 1 cm intervals, and the longest jump distance was calculated. The maximum value obtained in two trials was used as the standing broad jump score [13].

During the sit-and-reach measurement, the participants reached as far as possible with their arms rested on a specially designed device (TKK 5412, TAKEI, Tokyo, Japan) in a sitting posture with knees extended and whole legs on the ground. The score was the farthest distance reached in two trials [13].

### Body composition

Standing height was measured with a standing height meter in 0.5 cm increments, and weight was measured with an electronic scale in 0.1 kg increments. Body mass index (BMI) was defined as body mass/height^2^ (kg/m^2^). A multifrequency bioelectrical impedance analyzer, the InBody analyzer (430, Biospace, Seoul, Korea), was applied according to the manufacturer’s guidelines; this analyzer estimates body composition by differences in conductivity attributable to the different biological properties of each tissue. The body composition analyzer is based on a four-pole, eight-point contact electrode system that measures the impedance of the legs, trunk, and arms separately for each segment at three different frequencies (5, 50, and 250 kHz). Participants were measured in a rest posture and in a standing position with arms forward and elbows extended; InBody automatically provided body weight, body fat percentage, body fat percentage and skeletal muscle mass for each body segment. The skeletal muscle index (SMI; AMM/height^2^, kg/m^2^) was calculated by summing the two lower and two upper extremities (AMM) [10,14–16].

### Sarcopenia evaluation

A factory-calibrated hand dynamometer (TKK 5401, Takei, Tokyo, Japan) was used to assess the maximum voluntary isometric contraction of grip strength. All participants were instructed to grip the dynamometer with their right hand in an upright position with the arm next to the trunk and the elbow extended to 180°. The handle of the dynamometer was set to a size that was comfortable for the participant to hold (fits the second joint of each finger). Each participant completed two trials, and the highest value on each trial was used in the analysis [10,12,14–16].

The normal walking test was conducted on a 6 m course [14–16].

Calf girth has moderate to high sensitivity and specificity in predicting sarcopenia and low skeletal muscle mass. Therefore, maximal right calf girth was measured using a nonelastic tape according to the recommended protocol.

The Short Physical Performance Battery (SPPB) was conducted on participants according to the protocol of the National Institute on Aging. Tests were performed in the following order: (a) standing balance task, (b) gait task, and (c) standing chair task. Participants in the standing balance test were asked to maintain their posture with their feet in lateral parallel, semitandem, and tandem positions for 10 seconds. Scores ranged from 0 to 4 (maximum performance). A gait test was performed to determine the time required for the participant to walk 4 m at a normal pace. Utilizing a standard stable wooden chair (40 cm high, 90 cm wide, and 30 cm deep), participants performed five consecutive sit-to-stand tests from a seated position with their arms resting on their chest, and the time required was measured. In tests (a)–(c), the score for each category ranged from 0 to 4. The gait and sit-to-stand tests are based on previously established temporal quartiles in a large population, with the sum of the three components (a)–(c) providing the final SPPB score. The range is 0–12 points, with a score of 12 indicating the best lower extremity function [16–18].

### Statistical analyses

The results are presented as the mean ± standard deviation for all variables. All data were analyzed using JMP software (ver. 12.0 SAS Institute Inc., Tokyo, Japan). Nonparametric statistical analysis (Wilcoxon signed rank test) was applied to identify differences between the L-BW and NL-BW groups when the data were not normally distributed. Statistical significance was defined as *p*<0.05. NL-BW/L-BW effect sizes (ESs, Cohen’s d) for all datapoints were calculated using the following formula: ([NL-BW mean–L-BW mean]/low BW SD; d = 0.2, small effect; d = 0.5, moderate effect; and d = 0.8, large effect) [19].

## Results

A total of 18.6% of the participants were classified as the L-BW group, and 81.4% were classified as the NL-BW group. The NL-BW group had higher standing height (*p* = 0.01) and fat-free mass (*p* = 0.01) than the L-BW group (Table 1). No significant differences in fat mass were observed between the two groups for all body sites. Skeletal muscle mass was significantly greater (*p*<0.05) in the NL-BW group than in the L-BW group for all body sites. Effect sizes for the differences in skeletal muscle mass between the two groups were all larger in the NL-BW group than in the L-BW group (0.86–1.44) (Table 2). Knee extension muscle strength was higher in the NL-BW group than in the L-BW group (*p* = 0.04), but there were no differences between groups with respect to the other physical fitness evaluations (*p*>0.05) (Table 3). Except for SMI (*p* = 0.047), other sarcopenia evaluations did not differ between the two groups (*p*>0.05) (Table 4).

**Table 1:** The physical characteristics in young adult women.

Variable	Total	L-BW Group	NL-BW Group	Effect size
*N*	70	13	57	-
BW(g)	2849 (476)	2191 (421)	2999 (343) ^**^	1.92
Age (years)	18.5 (0.6)	18.3 (0.5)	18.5 (0.7)	0.42
Standing height (cm)	157.4 (5.4)	154.0 (3.2)	158.2 (5.6) *	1.30
Body weight (kg)	51.4 (6.7)	48.7 (5.5)	52.1 (6.9)	0.61
BMI (kg/m^2^)	20.8 (2.5)	20.6 (2.6)	20.8 (2.5)	0.08
Body fat (%)	26.7 (5.1)	27.5 (5.6)	26.5 (5.0)	0.18
Fat mass (kg)	14.0 (4.1)	14.3 (4.3)	14.0 (4.1)	0.08
Fat-free mass (kg)	20.3 (2.3)	18.9 (1.4)	20.6 (2.4) *	1.21

Data are given mean (standard deviation). Birth weight, BW. Body mass index, BMI. Low birth weight, L-BW. Not low birth weight, NL-BW. Skeletal muscle index, SMI. Effect size: L-BW Group vs. NL-BW Group. **p<0.01, vs. L-BW Group. *p<0.05, vs. L-BW Group.

**Table 2:** Body fat mass and skeletal muscle mass by region in young adult women.

Variable	Total	L-BW Group	NL-BW Group	Effect size
Fat mass				
Upper extremity fat mass (L), kg/m^2^	0.93 (0.29)	0.92 (0.29)	0.94 (0.30)	0.07
Upper extremity fat mass (R), kg/m^2^	0.91 (0.30)	0.92 (0.29)	0.91 (0.30)	0.02
Trunk fat mass, kg/m^2^	6.44 (2.28)	6.25 (2.32)	6.48 (2.29)	0.1
Lower extremity fat mass (L), kg/m^2^	2.33 (0.59)	2.28 (0.58)	2.34 (0.60)	0.11
Lower extremity fat mass (R), kg/m^2^	2.34 (0.59)	2.28 (0.58)	2.35 (0.60)	0.12
Skeletal muscle mass				
Upper extremity SMM(L), kg/m^2^	1.64 (0.28)	1.49 (0.21)	1.68 (0.28) *	0.86
Upper extremity SMM(R), kg/m^2^	1.69 (0.28)	1.55 (0.19)	1.72 (0.28) *	0.9
Trunk SMM, kg/m^2^	16.2 (1.8)	15.2 (1.23)	16.5 (1.8) *	1.02
Lower extremity SMM(L), kg/m^2^	5.75 (0.72)	5.25 (0.42)	5.86 (0.73) **	1.44
Lower extremity SMM(R), kg/m^2^	5.77 (0.70)	5.28 (0.44)	5.88 (0.71) **	1.36

Data are given mean (standard deviation). Birth weight, BW. Body mass index, BMI. Skeletal muscle mass, SMM. Low birth weight, L-BW. Not low birth weight, NL-BW. Light, L. R, Right. **p<0.01, vs. L-BW Group.

*p<0.05, vs. L-BW Group.

**Table 3:** The physical fitness assessment in young adult females.

Variable	Total	L-BW Group	NL-BW Group	Effect size
Knee extension (kg)	27.5 (5.0)	27.0 (5.1)	27.6 (5.0)	0.12
Standing broad jump (cm)	172 (16)	164 (15)	173 (16) *	0.65
Vertical jump (cm)	39.9 (5.5)	37.7 (5.4)	40.4 (5.5)	0.49
Sidestep test (reps/20 sec)	51.0 (6.4)	49.9 (6.8)	51.3 (6.4)	0.2
Sit-and-reach (cm)	50.9 (10.2)	50.7 (11.9)	50.9 (9.9)	0.02

Data are given mean (standard deviation). Birth weight, BW. Low birth weight, L-BW. Not low birth weight, NL-BW.

**p<0.01, vs. L-BW Group.

*p<0.05, vs. L-BW Group.

**Table 4:** The sarcopenia evaluations in young adult females.

Variable	Total	L-BW Group	NL-BW Group	Effect size
Grip strength (kg)	26.5 (4.8)	24.9 (3.5)	26.9 (5.0)	0.56
SMI (kg/m^2^)	5.98 (0.51)	5.72 (0.41)	6.03 (0.52) *	0.75
Thigh girth (cm)	48.5 (4.1)	48.3 (4.2)	48.6 (4.1)	0.05
Calf girth (cm)	34.5 (2.3)	33.8 (2.2)	34.7 (2.3)	0.42
SARC-F (unit)	0.5 (0.7)	0.6 (0.7)	0.5 (0.8)	0.11
SARC-Calf (unit)	3.7 (5.0)	4.8 (5.4)	3.5 (4.9)	0.24
Gait speed (m/sec)	1.48 (0.3)	1.50 (0.2)	1.48 (0.3)	0.12
Si-to-stand test (sec/5-time)	5.7 (1.0)	5.9 (1.0)	5.6 (1.0)	0.35
SPPB (unit)	12.0 (0.0)	12.0 (0.0)	12.0 (0.0)	-

Data are given mean (standard deviation). Birth weight, BW. Short physical performance battery, SPPB. Skeletal muscle index, SMI. Low birth weight, L-BW. Not low birth weight, NL-BW. **p<0.01, vs. L-BW Group. *p<0.05, vs. L-BW Group.

## Discussion

Based on the definition of the Japanese Ministry of Health, Labor and Welfare [20], one participant (960 g) had a birth weight of less than 1000 g (very low birth weight), 12 participants had a birth weight between 1500 g and 2500 g (low birth weight), 56 participants had a birth weight between 2500 g and 4000 g (normal birth weight), and one participant (4006 g) had a birth weight of more than 4000 g (high birth weight) in this study. Worldwide, health problems of low-birth-weight infants are often the focus of attention and are often investigated at 2500 g [20–23]. Therefore, in this study, it was decided to classify those who weighed less than 2500 g as the L-BW group and those who weighed 2500 g or more as the NL-BW group.

The standing height, weight, and grip strength (female: 157.4 cm, 51.4 kg, 26.7 kg) of the study participants were similar to the Japanese reference values for morphology and physical fitness (158.7 cm, 52.2 kg, 28.4 kg for 19-year-old females) [24]. Thus, the participants recruited for this study were truly representative of the general population of healthy young adult women in Japan. Previous study [25] suggests that it is important to maximize functional capacity of muscles in early life, which may be effective in preventing future bedridden (e.g., low sarcopenia incidence). Since N-BW had particularly low physical fitness of the lower limbs, it appears that L-BW infants need to take measures to prevent sarcopenia from an early stage, especially with a focus on improving lower limb muscle strength.

The physical fitness findings for young adult women showed no significant differences between the two groups in the four items, but standing long jump distance was significantly greater in the NL-BW group with a moderate effect size (Table 3). Standing long jump distance has been reported to be strongly correlated with 1 repetition maximum squat [26]. An examination of skeletal muscle mass by region showed that the NL-BW group was significantly higher than the L-BW group in all regions, but especially in lower extremity muscle mass, which had a greater effect size (Table 2). Thus, it can be inferred that differences in muscle mass, especially in the lower extremities, influenced standing long jump performance.

In this study, grip strength and SMI showed large effect sizes in the NL-BW group for the sarcopenia evaluation. In addition, the NL-BW group had a significantly higher SMI. This indicates that differences in birth weight are likely to make a major difference even in young adult women. Surprisingly, 53.8% of the L-BW group fell into the SMI <5.7 kg/m^2^ category, and almost twice as many participants compared with those in the NL-BW group (29.8%) had low skeletal muscle mass [15]. It has been reported in Europe and the United States that people born with low birth weight (birth weight <2500 g) are more susceptible to lifestyle-related diseases such as diabetes, hypertension, and cardiovascular disease [20–23]. Furthermore, L-BW is related with significantly lower muscle fiber scores at ages 68–76 [27]. Taken together, these studies and the results of the present study imply that L-BW infants may be expected to have a higher future incidence of sarcopenia than NL-BW infants. If an infant is born low weight, it may be important not only to prevent lifestyle-related diseases such as diabetes through appropriate weight control and nutritional management, but also to prevent sarcopenia through exercise guidance.

There were several limitations to this study. First, the participants were young Japanese women, which limited their ethnicity, age, and physical characteristics. Second, the low-birth-weight rate in this study was 18.6%, twice the average Japanese rate of 9.4%. Participants were recruited randomly, and thus the cause of this is unclear. Future robust studies with large sample sizes are needed to validate the present results. Third, the issue of sarcopenia from young adults to future sarcopenia is an issue that should be investigated, but currently no clear diagnostic criteria or cutoff values for young adults have been identified. Therefore, it was not possible to apply sarcopenia diagnostic criteria or cutoff values to young adult women in this study.

## Conclusion

L-BW female infants were shorter in standing height and smaller in skeletal muscle mass in terms of morphology at the time of young adulthood compared to NL-BW female infants. In addition, in terms of physical fitness, adult women with L-BW also had lower lower-limb power scores and a higher proportion of low skeletal muscle mass. Thus, it was suggested that low birthweight infants may be at risk of needing nursing care in old age (i.e., a high predicted incidence of sarcopenia) as well as thinness problems in the fertile generation.
